# Conversion to total hip arthroplasty after failed proximal femoral nail antirotations or dynamic hip screw fixations for stable intertrochanteric femur fractures: a retrospective study with a minimum follow-up of 3 years

**DOI:** 10.1186/s12891-017-1415-6

**Published:** 2017-01-25

**Authors:** Xianshang Zeng, Ke Zhan, Lili Zhang, Dan Zeng, Weiguang Yu, Xinchao Zhang, Mingdong Zhao

**Affiliations:** 1 Department of Orthopedics, The First Affiliated Hospital of Sun Yat-sen University, Huangpu East Road No. 183, Huangpu District, Guangzhou City, Guangdong Province 510700 China; 2 Department of Anesthesiology, The First Affiliated Hospital of Sun Yat-sen University, Huangpu East Road No. 183, Huangpu District, Guangzhou City, Guangdong Province 510700 China; 3 Ultrasonography Department, The First Affiliated Hospital of Sun Yat-sen University, Huangpu East Road No. 183, Huangpu District, Guangzhou City, Guangdong Province 510700 China; 4 Department of Orthopaedics, Jinshan Hospital, Fudan University, Longhang Road No. 1508, Jinshan District, Shanghai City, 201508 China

**Keywords:** Intertrochanteric fracture, Total hip arthroplasty, Complication, Proximal femoral nail antirotation, Dynamic hip screw

## Abstract

**Background:**

Conversion to total hip arthroplasty (CTHA) is a relatively common procedure after a failed dynamic hip screw (DHS) or proximal femoral nail anti-rotation (PFNA) fixation of intertrochanteric fractures, but there have been far fewer reports specifically describing the long-term results of CTHA after failed treatments of stable intertrochanteric fractures with DHS or PFNA. The aim of the present study was to compare the clinical and radiological outcomes of CTHA after failed PFNA or DHS fixations of stable intertrochanteric fractures after a minimum follow-up of 3 years.

**Methods:**

Between January 2005 and April 2014, we retrospectively reviewed 142 active elderly patients treated at our institution (a single institution study). A total of 72 patients (72 hips; 41 women, 31 men; mean age 76.9 years old; range 60–92 years old) who underwent conversion of a failed PFNA to a THA were compared with 70 patients (70 hips; 36 women, 34 men; mean age 75.0 years old; range 60–90 years old) who underwent CTHA after a failed DHS fixation. The mean follow-up periods were 48 (range 43–52) and 48 (range 44–52) months for the DHS and PFNA groups, respectively. Clinical and radiologic evaluations were performed on all patients. The primary outcome was the Harris Hip Score (HHS). The secondary outcomes were the complication rates.

**Results:**

The Harris Hip Score (HHS) improved from 50.61 ± 3.23 preoperatively to 85.28 ± 4.45 at the last follow-up in the PFNA group and from 51.46 ± 3.90 to 84.50 ± 4.34 in the DHS group, with no significant differences noted between the groups at each follow-up (P > 0.05). However, the complication rate in the converted DHS patients was significantly higher (42.9%) than that in the converted PFNA patients (20.8%; *P* = 0.003). Thirty-seven PFFs (2.4%) occurred during a mean follow-up of 44.4 months. The incidence of periprosthetic fractures was found to be significantly higher (*P* = 0.021) for the DHS group (15.7%) than for the PFNA group (4.2%).

**Conclusions:**

CTHA after failed DHS fixations of stable intertrochanteric fractures might be associated with a significantly higher complication rate than CTHA after failed PFNA fixations. Therefore, PFNA patients with stable intertrochanteric fractures may be more suitable for CTHA.

## Background

Intertrochanteric fractures are among the most important health care issues facing orthopaedic surgeons today [1, 2]. These fractures are well known to cause significant physical and functional impairment for patients and require substantial financial resources for both perioperative and rehabilitative care [3]. Proximal femoral nail anti-rotation (PFNA) and dynamic hip screw (DHS) fixation are accepted treatment options that are currently widely used as the primary treatment for stable intertrochanteric fractures (AO/OTA Type 3.1A1) [4, 5]. There is generally agreement that failed PFNA or DHS fixations of intertrochanteric fractures should be treated with a conversion to total hip arthroplasty (CTHA) whenever possible [3]. In China, an increasing incidence of intertrochanteric fractures has led to higher rates of CTHA [5].

Prior studies have reported that CTHA was a successful procedure that could be used to treat failed DHS or PFNA fixations of intertrochanteric fractures [3–5]. However, it has been unclear whether there are differences in the success rates for converting PFNA or DHS to a THA [3]. On the other hand, numerous studies have been published regarding comparisons of PFNA and DHS for the fixation of intertrochanteric fractures in active elderly patients [4–6]. Thus far, there has been no clear conclusion regarding the superiority of one of the approaches over the other. Some researchers hold that, for the management of stable intertrochanteric fractures, DHS should usually be recommended as the first choice and, in contrast, PFNA should be recommended for the treatment of unstable intertrochanteric fractures [4–8]. However, for stable intertrochanteric fractures, PFNA has also been shown to be an acceptable approach in extensive studies [4, 6]. In spite of some reports of good results, not all researchers have reported success with either DHS or PFNA [5, 7]. Thus, many active elderly patients with a failed DHS or PFNA are converted to a THA as a last option to salvage hip function. However, the failure rate for CTHA in active elderly patients remains high despite continuous improvements in the design and technique [6, 7]. With reported rates of dislocation greater than 22.5% and rates of periprosthetic fracture greater than 30% [9], it is very important to continue to monitor the clinical and radiological outcomes and to improve the treatment strategies for failed DHS or PFNA-II fixations of stable IFFs to reduce the complications in these patients [4–6]. In addition, the results of CTHA after failed DHS or PFNA are still controversial, although some studies have reported no differences [9–11]. To date, none of the previous reports have directly compared the clinical and radiological outcomes of CTHA after failed DHS or PFNA fixations of stable intertrochanteric fractures.

The aim of the present study was to compare the clinical and radiological outcomes of CTHA after failed PFNA or DHS fixations of stable intertrochanteric fractures after a minimum follow-up of 3 years.

## Methods

Between January 2005 and April 2014, 192 patients (192 hips) underwent CTHA following failed treatment of stable intertrochanteric fractures with DHS or PFNA devices at our institution (a single centre). The average time interval from the initial fracture fixation (PFNA or DHS) to CTHA fixation was 11 (range 3–16) months. The inclusion criteria were: active elderly patients aged 60–92 years old; a prior intertrochanteric fracture (type AO/OTA 31. A1); failed fixation due to screw cut-out, nonunion, avascular necrosis or insufficient initial fixation; normal cognitive function; ability to walk independently without aids before fracture; and eligibility to receive a standard CTHA device (Standard-device, Stryker, Mahwah, New Jersey). The exclusion criteria were: pathological fractures, metastatic disease, infection, neoplasia, arthritis, ASA score V, ipsilateral lower-limb surgery, contralateral hip fracture or other revision procedures. Based on these criteria, 29 patients were excluded. Another 16 refused to participate, leaving 147 patients eligible for the study. The patient characteristics are expressed as the means with SDs or as frequencies and percentages. The primary outcome was the clinical outcome, as defined by the Harris Hip Score (HHS), which was collected at 1 h preoperatively and post-operatively at 2, 6, 12, 24, 36 months and at the last follow-up. The secondary outcomes were the incidence and distribution of complications.

### Surgical methods

All of the patients were treated with CTHA by the surgeons (WY, XCZ, XZ and KZ) after failed fixation (PFNA or DHS) for stable intertrochanteric fractures. All of the operations were carried out under general anaesthesia. Two surgical approaches for conversion to a THA were performed according to standard protocols for CTHA, which were recommended by the manufacturers and have been previously described in the literature [11, 12]. All of the CTHAs were carried out through a posterolateral approach with the patient in a lateral decubitus position. The previous DHS or PFNA was removed. With the hip joint dislocated, osteotomy of the femoral neck was performed with an oscillating saw. After removal of the femoral head, the acetabulum was reamed [13]. The details of each surgical approach are almost identical. Immediate postoperative radiographs and ultrasonic examination were used to evaluate the quality of operation. All of the patients underwent a clinical evaluation to determine the HHS. Intra- and post-operative complications were recorded.

Fisher’s exact test and the chi-square test were utilized for categorical variables and numerical variables, respectively. All of the reported *P* values were two-sided, and a *P* value < 0.05 was considered to be significant for all of the statistical tests. All hips were assumed to be independent in the statistical analysis. All statistical analyses were performed using the SPSS software program (version 22.0, IBM Inc. Chicago, Illinois).

## Results

**Fig. 1:**
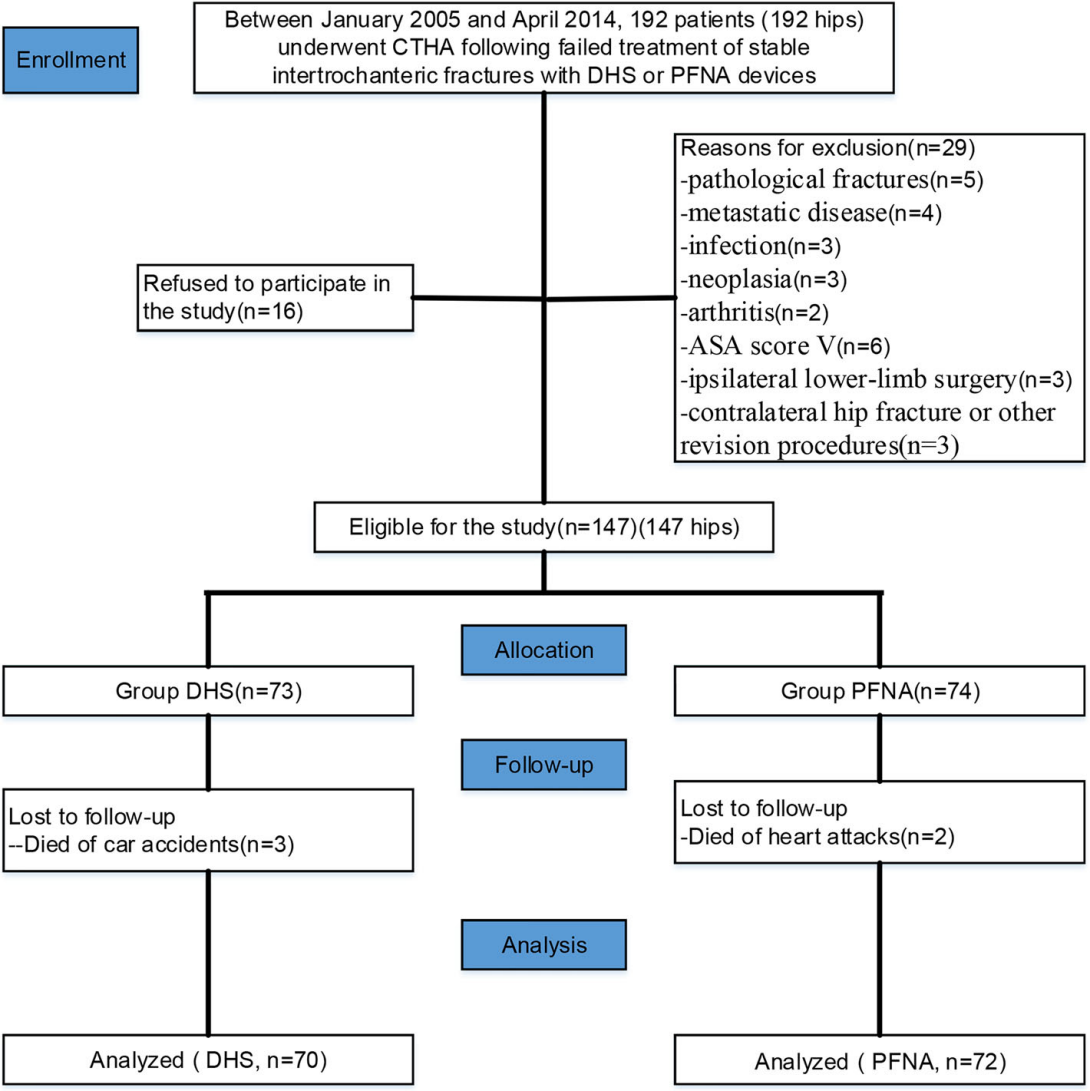
Flow diagram demonstrating methods for identification of studies to assess the treatment of CTHA after failed PFNA or DHS fixations of stable intertrochanteric fractures in the elderly

During the follow-up period, 3 patients in the PFNA group died in car accidents, and 2 patients from the DHS group died of heart attacks. Therefore, 142 patients (142 secondary operations, all were CTHAs) were included in the final analysis and their records were retrospectively reviewed (2 groups: DHS [*n* = 70] and PFNA [*n* = 72]) (Fig. 1). The patient demographics are shown in Table 1. None of 142 patients were lost to follow-up, and all 142 patients were available for review (zero mortality). Sixty-eight male patients and 74 female patients were evaluated in the current study. The PFNA group had 31 (43.1%) men and 41 (56.9%) women. The DHS group had 34 (48.6%) men and 36 (51.4%) women. There were no significant differences between the groups regarding sex, the modes of prior failed fixation, ASA score, AO/OTA classification, BMI, initial complication rates before the CTHA procedure, length of stay in the hospital, intraoperative blood loss, length of the operation or preoperative HHS. The average age at the CTHA procedure was 76.93 years old (range, 60–92 years; SD, 9.25) in the PFNA group and 74.96 years old (range, 60–90 years; SD, 8.59) in the DHS group (*P* = 0.19). There were no significant differences in the medical complications between groups (*P* = 0.442) with 3 of 72 (4.2%) patients affected in the PFNA group and 5 of 70 (7.1%) patients affected in the DHS group (Table 3).

**Table 1:** Patient demographics in the groups with different implant types

Variable	PFNA (*n* = 72)	DHS (*n* = 70)	*P* value
Age (years)	76.93 ± 9.25	74.96 ± 8.59	0.19
Sex (M:F)	31:41	34:36	0.51
Modes of prior failure fixation			0.94
Screw cut-out	24	23	0.95
Nonunion	21	18	0.65
Avascular necrosis	16	16	0.93
Insufficient initial fixation	11	13	0.60
ASA score, No.			0.82
1	10	12	0.59
2	18	17	0.92
3	22	24	0.64
4	22	17	0.40
Femoral fixation (NO.)			0.41
Cemented	30 (41.7%)	34 (48.6%)	
Uncemented	42 (58.3%)	36 (51.4%)	
Length of follow-up (months)	47.92 ± 4.31	48.10 ± 4.12	0.80
Body Mass Index (BMI, kg/m2)	29.50 ± 2.70	28.90 ± 1.50	0.10
AO/OTA classification (NO.)			0.88
3.1A1.1	12	13	0.77
3.1A1.2	37	33	0.61
3.1A1.3	23	24	0.77

### Clinical outcomes

There was an improvement in the HHS between the preoperative evaluation and the last follow-up in both groups (Table 2). The mean lengths of follow-up were 47.92 months (interquartile range [IQR] 43 to 52) and 48.10 months (IQR 44 to 52) for the PFNA and DHS groups, respectively.

**Table 2:** Comparison of the Harris Hip Scores at 1 h preoperatively; 2, 6, 12, 24 and 36 months postoperatively; and at the last follow-up

	PFNA	DHS	*p*
1 h Pre-operatively	50.61 ± 3.23	51.46 ± 3.90	0.16
2 Months Postoperatively	78.33 ± 1.65	77.90 ± 1.77	0.13
6 Months Postoperatively	79.31 ± 2.09	78.53 ± 2.98	0.08
12 Months Postoperatively	82.54 ± 2.49	81.91 ± 4.36	0.30
24 Months Postoperatively	86.35 ± 4.38	85.03 ± 4.47	0.78
36 Months Postoperatively	86.14 ± 3.22	85.30 ± 3.72	0.15
Last follow-up	85.28 ± 4.45	84.50 ± 4.34	0.29

The HHS in the PFNA group improved significantly from 50.61 ± 3.23 to 85.28 ± 4.45, and that in the DHS group improved significantly from 51.46 ± 3.90 to 84.50 ± 4.34. There were no significant differences between groups in terms of the HHS at the last follow-up (*P* = 0.29).

### Radiological outcomes

Postoperative radiographs were available for all patients, with a mean follow-up of 48 months (43–52 months). No intra-operative fractures were observed in either group. There were no significant differences in the rate of periprosthetic fracture between cemented and uncemented implants.

The complication rate in the PFNA group was 16.7% (12/72) compared with 37.1% (26/70) in the DHS group. Three (4.2%) periprosthetic fractures occurred. There was a statistically significant difference regarding the rate of periprosthetic fractures between the groups. Ten complications in 10 patients were observed in the PFNA group, including postoperative periprosthetic fractures, prosthetic instability, dislocation, limb length discrepancy (>2.5 cm), abductor tendon deficiency, Brooker class 6 heterotopic ossification, and aseptic loosening (Table 3).

**Table 3:** Complications of conversion to total hip arthroplasty

Variable	PFNA (*n* = 72)	DHS (*n* = 70)	*P* value
Total complications	15	31	0.003
Patients affected	15 (20.8%)	31 (42.9%)	0.003
Medical complications	3	5	0.442
Patients affected	3 (4.2%)	5 (7.1%)	0.442
Urinary tract infection	1	1	1.000
Pulmonary embolism	0	2	0.241
Atrial fibrillation	2	0	0.497
Acute renal failure	0	2	0.241
Orthopaedic complications	12	26	0.011
Patients affected	12 (16.7%)	26 (37.1%)	0.011
Periprosthetic fracture	3 (4.2%)	11 (15.7%)	0.021
Prosthetic instability	1	2	0.617
Dislocation	1	3	0.363
Limb length discrepancy (>2.5 cm)	1	2	0.617
Abductor tendon deficiency	2	2	1.000
Heterotopic ossification	1	1	1.000
Intraoperative nerve injury	1	1	1.000
Aseptic loosening	0	1	0.241
Periprosthetic infection	2	2	1.000

In the DHS group, orthopaedic complications were significantly more common, at a rate of 37.1%. Twenty-four complications were identified in 24 patients, including postoperative periprosthetic fractures, prosthetic instability, dislocation, limb length discrepancy (>2.5 cm), Brooker class 6 heterotopic ossification, aseptic loosening and late deep infection requiring removal of hardware. Eleven (15.7%) periprosthetic fractures occurred. More than one-third (37.1%; 26/70) of the patients had experienced an orthopaedic complication by the last follow-up in the DHS group compared with a 16.7% (12/72) orthopaedic complication rate in the PFNA group (*P* = 0.003) (Table 3).

## Discussion

Most retrospective studies have investigated intertrochanteric fractures in a comprehensive manner. However, most follow-up period is commonly reduced to 2-years post-operation, and the implants are often compared in terms of their use [8, 11, 12]. Consequently, these studies had substantial differences compared with our study in terms of the inclusion criteria and the parameters investigated.

The evidence in the literature regarding the optimal method that should be used for the initial internal fixation of stable intertrochanteric fractures is inconclusive [8, 11, 12, 14]. Prior evidence has suggested that DHS may be superior to PFNA, but these findings were based on studies with small sample sizes and low event numbers [15]. Moreover, in China, 70% of surgeons prefer PFNA over DHS for treating a stable intertrochanteric fracture in active elderly patients [10]. Other studies have previously reported that DHS was the best choice for the initial treatment of stable intertrochanteric fractures [6, 7, 9, 16, 17]. Although the complication rates of DHS devices ranged from 12 to 34% [10], DHS was still regarded as a preferred device for stable intertrochanteric fractures in active elderly patients. However, another problem that must be considered is that the results of CTHA after a failed DHS fixation compared to a failed PFNA fixation are relatively controversial [7, 8, 16–18]. There is currently no consensus about which type of implant to use or what technique to perform in patients with stable intertrochanteric fractures previously treated with DHS fixation or PFNA. Some studies reported no difference in the clinical and radiological outcomes [7–12, 19, 20], while others reported higher complication rates and revision rates for one approach after CTHA [17, 21]. To address this controversy, we compared the clinical and radiological outcomes of CTHA after a failed PFNA or DHS fixation for stable intertrochanteric fractures.

There have only been a few previous reports that have focused on CTHA after a failed PFNA or DHS fixation for stable intertrochanteric fractures. Furthermore, the majority of these studies had low numbers of patients and short follow-up periods, and therefore, drawing conclusions about the relative superiority of one implant over the other is inappropriate. Unnanuntana et al. [22] retrospectively reviewed 78 patients who underwent CTHA after failed DHS fixations of stable intertrochanteric fractures and found that orthopaedic complications were more frequent. Diwanji et al. [9] performed 163 CTHAs after failed DHS fixations of a prior stable intertrochanteric fracture and noted a 14.1% (23/163) orthopaedic complication rate, including 11 dislocations (6.7%) and 12 femur fractures (7.4%). In the current study, a 37.1% orthopaedic complication rate (42.9% total complication rate) was identified in the DHS group compared with a 16.7% orthopaedic complication rate (20.8% total complication rate) in the PFNA group. The present complication rate in the DHS group was comparable to those of other series using similar osteosynthesis techniques.

In the previous study [3, 8, 19, 23], the incidence of periprosthetic fractures after CTHA ranged from 11.9% to 28%. The incidence of periprosthetic fractures in these studies was higher in comparison with the results of our study (14 out of 142, 9.9%). A recent study has reported the incidence of periprosthetic fractures after CTHA was used to treat failed PFNA or DHS fixations of intertrochanteric fractures in a large cohort [12]. Fifty-two of the 594 patients (8.8%) sustained a periprosthetic fracture during a mean follow-up of 4 years, and 71% of the fractures occurred within 1 year. The incidence of periprosthetic fractures in that study was consistent with the results of our study. In this study, prior DHS-treated patients receiving CTHA tended to have less resistance to periprosthetic fractures. One of the major reasons for this finding might be that the patients sustaining stable intertrochanteric fractures treated with a DHS tended to have poorer bone quality caused by stress shielding, which was in line with the prior consensus that a high incidence of mechanical complications (periprosthetic fractures) was recorded in patients who had received prior DHS treatment [7, 9, 16, 24]. As demonstrated by recent biomechanical testing [19], patients undergoing DHS may have poorer bone quality caused by stress-shielding than patients undergoing PFNA, which might partly explain the destruction of the bone and the disuse and atrophy of the proximal femur.

Both cemented and uncemented CTHA designs are being successfully used for the treatment of failed DHS fixations of stable intertrochanteric fractures, despite the fact that uncemented CTHAs are commonly offered to youthful patients [16]. Moreover, previous studies had evaluated the outcome of the cemented and uncemented CTHA, and reported no difference between cemented and uncemented CTHA was observed with respect to stem performance [2, 22, 24]. That was consistent with our conclusion.

A growing but still very limited body of literature has shown that conversion from prior PFNA fixation is better compared with conversion from prior DHS fixation with regard to the postoperative HHS after 0.5–1.5 years of follow-up [8, 15, 17, 25]. However, similar to a multi-centre, randomized study [26], we found no obvious differences in the postoperative HHS between the groups treated with the two types of conversions after a median of 3 years of follow-up. An obvious explanation for this may be the differences in the follow-up period compared to other studies. In addition, our results were also in line with other previous studies based on data acquired from the Healthcare Cost and Utilization Project’s Nationwide Inpatient Sample [13, 21, 23, 27].

This study should be interpreted in light of important limitations. First, this study is observational and it is possible that we failed to address every potential confounding variable in our analyses. Second, it is a retrospective study with all the problems inherent with this methodology. Third, because the conversion often occurred several years after the initial treatment with PFNA or DHS, the initial fracture pattern of stable intertrochanteric fractures may not have been recorded. It is possible that the fractures treated with DHS may have been more complex than those treated with PFNA, which might have eventually led to more malunions and fractures in the DHS group, thus making intra- and post-operative complications more common than in randomized, controlled cohorts.

## Conclusions

We found significant complication rates associated with the conversion of both DHS and PFNA to THA. Moreover, CTHA after a failed DHS was associated with a higher complication rate. Thus, prior PFNA-treated patients with stable intertrochanteric fractures appear to be more suitable for treatment with CTHA. Further follow-up is needed to confirm whether these results persist over a longer term.

## Abbreviations

CTHA: Conversion total hip arthroplasty
DHS: Dynamic hip screw
HHS: Harris hip score
IQR: Interquartile range
PFNA: Proximal femoral nail anti-rotation
SD: Standard deviation
